# An App-Based Digit Symbol Substitution Test for Assessment of Cognitive Deficits in Adults With Major Depressive Disorder: Evaluation Study

**DOI:** 10.2196/33871

**Published:** 2022-10-27

**Authors:** Roger S McIntyre, Orly Lipsitz, Nelson B Rodrigues, Mehala Subramaniapillai, Flora Nasri, Yena Lee, Ben Fehnert, James King, Lambros Chrones, Kevin Kratiuk, Sharif Uddin, Joshua D Rosenblat, Rodrigo B Mansur, Maggie McCue

**Affiliations:** 1 University Health Network Mood Disorders Psychopharmacology Unit Toronto, ON Canada; 2 Brain and Cognition Discovery Foundation Toronto, ON Canada; 3 Department of Psychiatry University of Toronto Toronto, ON Canada; 4 Canadian Rapid Treatment Center of Excellence Mississauga, ON Canada; 5 Ctrl Group London United Kingdom; 6 Cognition Kit Cambridge United Kingdom; 7 Takeda Pharmaceuticals U.S.A., Inc. Lexington, MA United States

**Keywords:** depression, DSST, Digit Symbol Substitution Test, smartphone, technology, measurement-based care, cognition

## Abstract

**Background:**

Cognitive dysfunction is an impairing core symptom of depression. Among adults with major depressive disorder (MDD) treated with antidepressants, residual cognitive symptoms interfere with patient-reported outcomes. The foregoing characterization of cognitive symptoms provides the rationale for screening and assessing the severity of cognitive symptoms at point of care. However, clinical neurocognitive assessments are time-consuming and difficult, and they require specialist expertise to interpret them. A smartphone-delivered neurocognitive test may offer an effective and accessible tool that can be readily implemented into a measurement-based care framework.

**Objective:**

We aimed to evaluate the use of a smartphone-delivered app-based version of the established Cognition Kit Digit Symbol Substitution Test (DSST) neurocognitive assessment compared to a traditional paper-and-pencil version.

**Methods:**

Convergent validity and test-retest reliability of the 2 versions were evaluated. Patient satisfaction with the app was also assessed.

**Results:**

Assessments made using the app-based Cognition Kit DSST were highly correlated with the standard paper-and-pencil version of the test, both at the baseline visit (*r*=0.69, *df*=27; *P*<.001) and at the end-of-study visit (*r*=0.82, *df*=27; *P*<.001), and they were positively evaluated by 30 patients as being user-friendly, easy to navigate, and preferable over the paper-and-pencil version of the DSST. However, although the app-based Cognition Kit DSST was validated in patients with MDD, it still needs to be evaluated in healthy controls.

**Conclusions:**

App-based DSST may facilitate a more personalized, convenient, and cost-effective method of cognitive assessment, helping to guide measurement-based care and psychotherapeutic and pharmacologic treatment options for patients with MDD.

**Trial Registration:**

ClinicalTrials.gov NCT03999567; https://tinyurl.com/2p8pnyv7

## Introduction

Major depressive disorder (MDD) is an episodic illness characterized by a persistently depressed mood or loss of interest in activities that causes significant impairment in daily life [1,2]. Cognitive impairment is a core domain disturbance in MDD, with disturbances in cognitive function being listed among the criteria that define a major depressive episode [2].

Approximately 90%-95% of adults with MDD experience cognitive symptoms during a depressive episode [3], including impairments in executive function, attention, learning and memory, and processing speed [4,5]. The foregoing cognitive deficits have been demonstrated to mediate social, functional, and work-related disability associated with MDD and have a long-term impact that often persists when depressive symptoms have abated [5,6]. Indeed, during periods of remission, approximately half of all depressed patients will continue to experience cognitive deficits, which presage patient-reported outcomes (PROs) in adults with MDD [3,7].

Addressing cognitive symptoms can be a clinical priority when treating some patients with MDD, particularly those whose MDD appears to be significantly impairing their daily functioning and treatment has failed to resolve these symptoms [8]. Impaired cognitive functioning is also progressive in some patients with MDD [5,9], and there is evidence to suggest that cognitive function deteriorates further with each major depressive episode [4]. Accordingly, there is a need to address cognitive dysfunction in patients with MDD, as it substantially interferes with daily psychosocial functioning and can lead to adverse long-term outcomes. For example, patients with MDD may have poorer workplace performance, which is a result of impaired cognitive functioning [5,6,10]. Cognitive deficits, therefore, influence PROs and reduce individuals’ quality of life and functioning [11].

The critical and pervasive role that cognitive deficits play in the functioning PROs in patients with MDD invites the need for accessible, convenient, and effective measurement-based care (MBC) assessment tools that offer more than a simple evaluation of the presence or absence of symptoms. Tools that collect valuable information about symptoms and potential changes that could impact general well-being may provide greater insight into a patient’s condition, supporting individualized care that can improve overall treatment outcomes. Guiding principles of MBC are more likely to be implemented at the point of care as tools that are brief and patient administered, provide actionable information, and are preferably digitalized in keeping with busy office practices [12].

Many commonly used comprehensive neurocognitive tests are effective MBC assessment tools, but they are lengthy and cumbersome to administer and complete and often require professional interpretation, limiting their implementation outside of a clinical environment [13,14]. Web-based tools integrate both subjective and objective measures of cognition, are typically free of charge for the patient, digitalized, implemented remotely (ie, using a tablet), and require less time to complete (ie, approximately 10 minutes) [12,15]. However, web-based tools may not be accessible for all patients if a paid software subscription is required or the test has not been optimized for smartphone delivery and must be delivered using a computer or tablet with a large screen [16]. Recent progress in smartphone technology and mobile apps presents a unique prospect in this scenario. Several health-related smartphone apps have already been implemented in other chronic diseases (eg, diabetes mellitus), where it has been shown to be acceptable to end users, provide actionable data, and facilitate health outcomes [17]. The ubiquity of smartphones provides an opportunity to screen and measure the presence of cognitive functions in patients with MDD via smartphone-based neurocognitive assessments. Similar to web-based tools, smartphone-based neurocognitive assessments can also be free of charge for the patient, easy to administer, and may require even less time to complete.

The Digit Symbol Substitution Test (DSST) is an MBC assessment tool that provides multidomain assessment of neurocognitive functions and has been extensively validated in psychiatric, medical, and general populations [16]. This study was designed to evaluate an app-based Cognition Kit DSST as a screening and assessment tool for cognition in MDD that can be delivered via a smartphone.

## Methods

### Ethics Approval

This study was approved by the institutional review board of Advarra (Pro00037042) prior to initiation of the study. All participants provided written informed consent prior to enrollment.

### Study Design and Participants

Patients enrolled at the Canadian Rapid Treatment Center of Excellence were approached and asked to participate. Adult patients (aged 18-65 years) experiencing a moderate-to-severe major depressive episode (based on the Montgomery-Åsberg Depression Rating Scale [MADRS] with a total score ≥20) in the context of MDD were enrolled in this prospective, longitudinal validation study (Clinical Trial Identifier NCT03999567). Patients were not under treatment during participation and could not have had a change in their medication up to 2 weeks prior to participation. Participants were recruited by 2 research coordinators who were not responsible for patient care at the center.

The diagnosis of MDD was ascertained clinically and confirmed using the Mini-International Neuropsychiatric Interview (MINI). Patients were excluded if they had a comorbid psychiatric condition primary to MDD, used benzodiazepines within 12 hours of cognitive assessment, consumed alcohol within 8 hours of cognitive assessment, or used marijuana in an inconsistent or abusive manner. Patients were also excluded if they had current alcohol or substance use disorder confirmed by the MINI; physical, cognitive, or language impairments; diagnosed reading disability; dyslexia; or a clinically significant learning disorder. Use of electroconvulsive therapy in the past 6 months or a history of moderate-to-severe head trauma, neurological disorders, or unstable medical conditions that affect the central nervous system were criteria for exclusion from the study. If patients had a previous history or were currently experiencing symptoms of mania or hypomania or had a history of seizures and epilepsy, they were not eligible to participate. Patients were asked not to change medications 1 week prior to the baseline study visit and in the week between the baseline and end-of-study visits.

### Procedure

**Figure 1:**
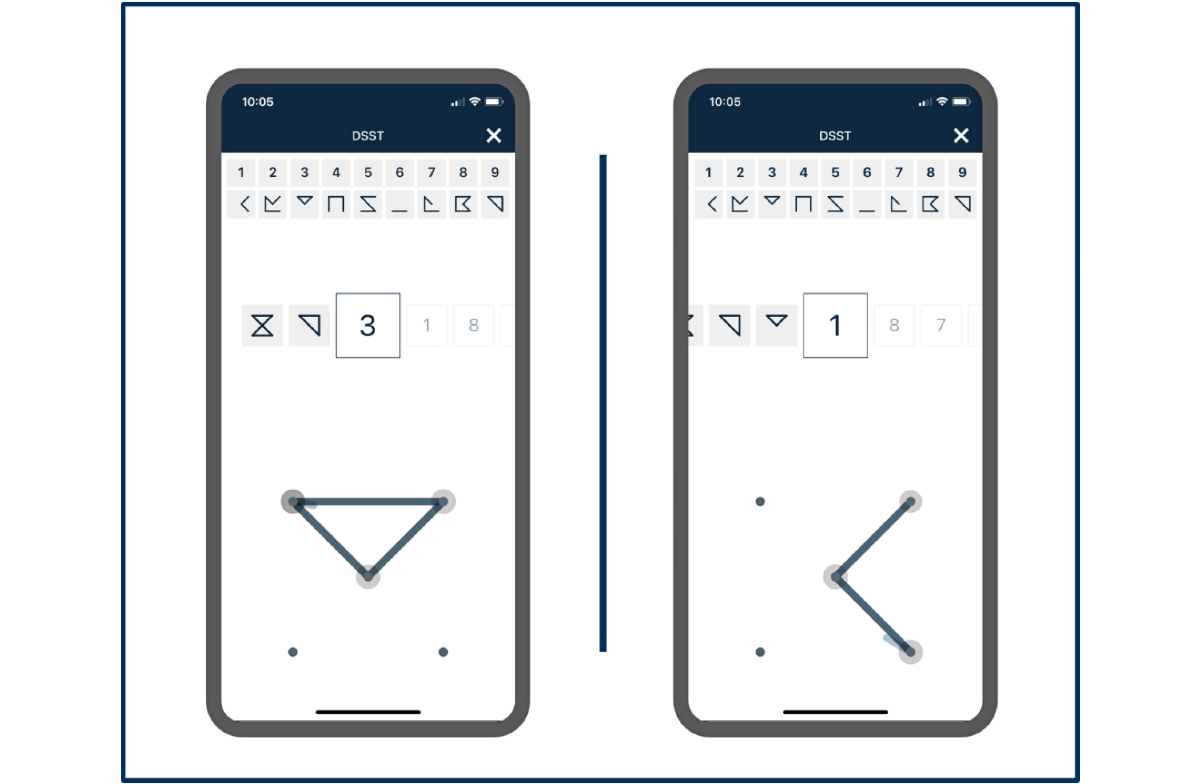
App-based Cognition Kit Digit Symbol Substitution Test (DSST) screenshots.

Participants completed both the app-based Cognition Kit DSST (Cognition Kit Ltd; Figure 1) on an Apple iPhone with finger-screen interaction and the paper-and-pencil DSST at 2 study visits: first at the baseline visit and then 1 week later at the end-of-study visit. The order of the app-based and paper-and-pencil assessments was counterbalanced between the 2 visits and between study participants. The DSST was based on the Wechsler Adult Intelligence Scale–Revised version [16]. Data from each app-based Cognition Kit DSST was stored in a system compliant with the Health Insurance Portability and Accountability Act to ensure data privacy.

Overall, depressive symptom severity (based on MADRS), consummatory anhedonia (based on Snaith-Hamilton Pleasure Scale [SHAPS]) [18], and anxiety (based on Hamilton Anxiety Rating Scale [HAM-A]) [19] were assessed at each visit. At the end-of-study visit, patients completed a 10-item app satisfaction survey. Each item of this survey was scored on a 5-point Likert scale, where 1 indicated “strongly disagree” and 5 indicated “strongly agree.”

Convergent validity of the app-based Cognition Kit DSST versus the paper-and-pencil DSST and the test-retest reliability of each instrument were assessed by calculating the Pearson correlation coefficient (partial correlation) using SPSS (version 23.0; IBM Corp), controlling for age.

## Results

### Patient Demographic and Clinical Characteristics

**Figure 2:**
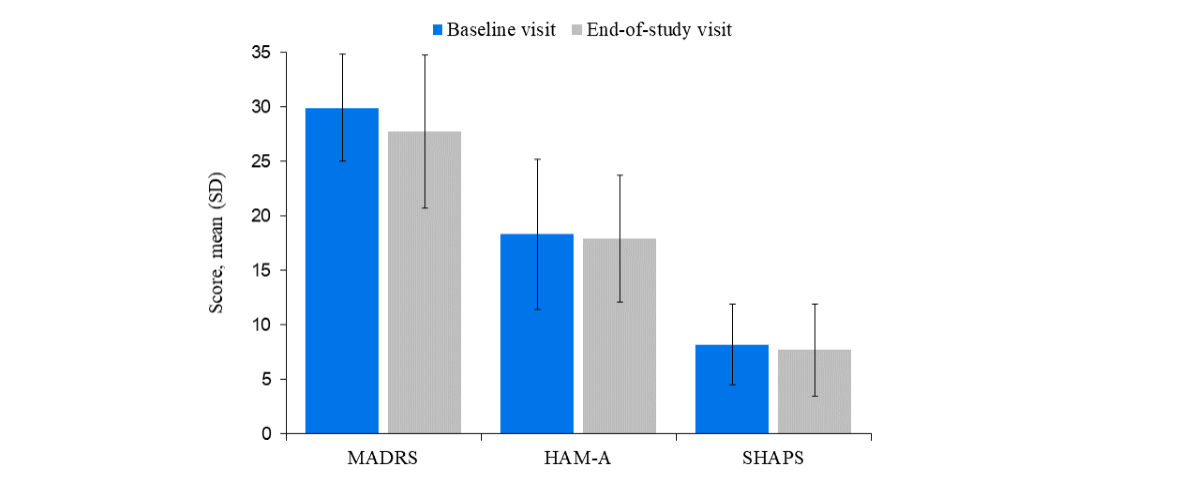
Clinical measures at baseline and end-of-study (N=30). HAM-A: Hamilton Anxiety Rating Scale; MADRS: Montgomery-Åsberg Depression Rating Scale; SHAPS: Snaith-Hamilton Pleasure Scale.

Prescreening was performed for 47 potential patients, of which a total of 30 patients were eligible for inclusion (Multimedia Appendix 1). All patients completed both study visits; 17 (57%) were female, and the mean age was 42 (SD 13) years. Approximately two-thirds (19/30, 63%) of patients had completed a college or university education. At baseline, 57% (17/30) of patients were currently taking an antidepressant medication (Table 1). Patients had a mean MADRS total score of 29.9 (SD 4.9) at baseline. Mean MADRS, HAM-A, and SHAPS scores did not differ greatly between the baseline and end-of-study visits (Figure 2).

**Table 1:** Patient demographic and clinical characteristics at baseline.

Demographics or clinical characteristics		All study participants (N=30)
Age (years), mean (SD)		42 (13)
**Sex, n (%)**		
	Female	17 (57)
	Male	13 (43)
**Race, n (%)**		
	White	25 (83)
	Asian	2 (6.7)
	Multiracial	2 (6.7)
	Latin American	1 (3.3)
**Highest level of education completed,** **n** **(%)**		
	High school	7 (23)
	College or university	19 (63)
	Graduate school	4 (13)
**Antidepressant medication**		
	Taking antidepressant medication, n (%)	19 (63)
	Current antidepressants, mean (SD)	1.0 (0.98)
	Lifetime antidepressants^a^, mean (SD)	7 (7)

^a^Number of antidepressants used throughout the patient’s lifetime.

### Convergent Validity

**Figure 3:**
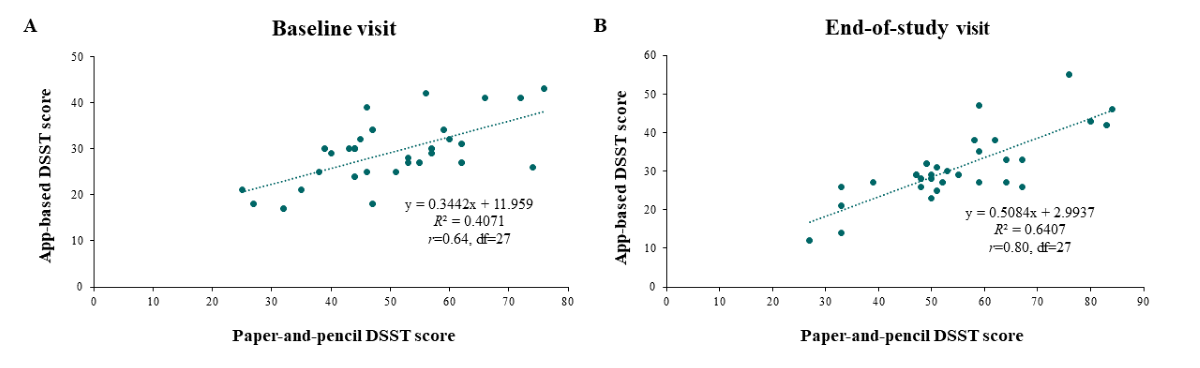
Correlation between app-based DSST and paper-and-pencil DSST scores at (A) baseline visit and (B) end-of-study visit (N=30). The correlation is not adjusted for age. DSST: Digit Symbol Substitution Test.

Patients had mean DSST scores of 29 (SD 7) and 50 (SD 13) at baseline for the app-based and paper-and-pencil DSST, respectively. At the end of the study, the mean DSST scores were 31 (SD 9) and 55 (SD 14), respectively, for the app-based and paper-and-pencil DSST. The app-based Cognition Kit DSST and the paper-and-pencil DSST were positively correlated at both the baseline visit (*r*=0.64, *df*=27; Figure 3A) and the end-of-study visit (*r*=0.80, *df*=27; Figure 3B). The corresponding partial correlations after adjusting for age were *r*=0.69 (*df*=27; *P*<.001) and *r*=0.82 (*df*=27; *P*<.001) at baseline and end-of-study visits, respectively. Differences in the Cognition Kit DSST score from the baseline visit to the end-of-study visit versus the corresponding differences in the paper-and-pencil DSST score trended toward positive correlation (*r*=0.24, *df*=27; *P*=.21).

### Test-Retest Reliability

**Figure 4:**
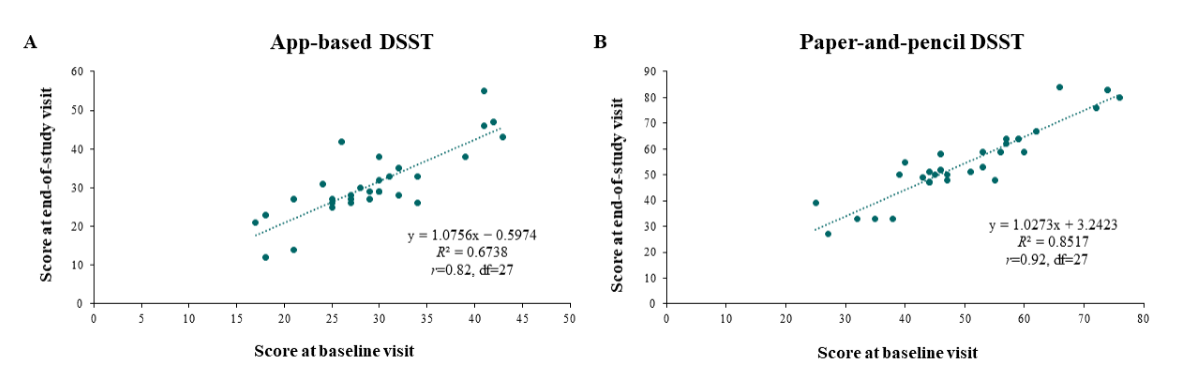
Test-retest reliability of (A) app-based DSST and (B) paper-and-pencil DSST (N=30). Results are not adjusted for age. DSST: Digit Symbol Substitution Test.

A positive correlation was found between scores at the baseline visit and scores at the end-of-study visit for both the app-based Cognition Kit DSST (*r*=0.82, *df*=27; Figure 4A) and the paper-and-pencil DSST (*r*=0.92, *df*=27; Figure 4B). The corresponding partial correlations after adjusting for age were *r*=0.75 (*df*=27; *P*<.001) and *r*=0.92 (*df*=27; *P*<.001) for the app-based Cognition Kit DSST and the paper-and-pencil DSST, respectively.

### Cognition Kit DSST App Satisfaction

**Figure 5:**
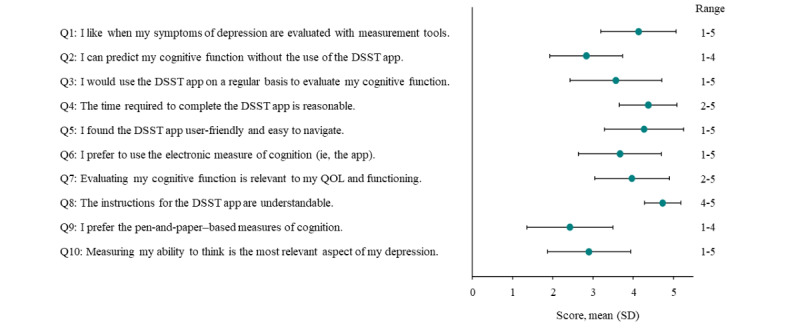
App satisfaction survey mean scores (N=30). Each question was scored on a 5-point Likert scale where 1=strongly disagree and 5=strongly agree. DSST: Digit Symbol Substitution Test; QOL: quality of life.

In the app satisfaction survey, 87% (26/30) of patients reported that they agreed or strongly agreed that the Cognition Kit DSST app was user-friendly and easy to navigate. Overall, patients reported the highest levels of agreement with the following statements, each of which received a mean score >4 on the 5-point Likert scale (where a score of 4 indicated “agree” and a score of 5 indicated “strongly agree”): “I like when my symptoms of depression are evaluated with measurement tools”; “The time required to complete the DSST app is reasonable”; “I found the DSST app user-friendly and easy to navigate”; and “The instructions for the DSST app are understandable” (Figure 5). Additionally, 57% (17/30) of patients reported that they preferred to use the Cognition Kit DSST over the paper-and-pencil DSST.

## Discussion

### Principal Results

The Cognition Kit DSST was capable of detecting cognitive deficits in adults with MDD. The app-based DSST was highly correlated with the standard paper-and-pencil version of the test and was positively evaluated by 30 patients as being user-friendly, easy to navigate, and preferable to the paper-and-pencil version of the DSST. The change in mean DSST scores from baseline to end of the study observed in both the paper-and-pencil and app-based DSST may be due to intervention effects, random effects, regression to mean, or practice effects [16,20].

### Comparison With Prior Work

Antidepressant therapy may relieve depressive symptoms, but resolution of these symptoms is not well correlated with functional recovery and quality of life, which are often higher priority outcomes for patients with MDD [21]. Therefore, regular assessment of functional outcomes using validated measures, such as the DSST, may assist health care professionals in optimizing treatment for patients with MDD [21].

MBC has been highly evaluated in clinical practice in adult patients with MDD [22], but uptake remains low (<20%) in mental health settings [21]. Therefore, it is important that a convenient and accessible method of assessment is made available to patients with MDD, especially those who may be experiencing a degree of cognitive impairment. Indeed, the National Institute of Mental Health’s public health trial—Sequenced Treatment Alternatives to Relieve Depression—demonstrated the usefulness of MBC for guiding psychotherapeutic and pharmacologic treatment approaches in patients with depression [22]. Strategies to reduce barriers to MBC and appropriately implement it as part of routine practice should be prioritized.

Neurocognitive tests via smartphones offer an effective and accessible tool that can be readily implemented into an MBC framework. In particular, individual tests such as the Cognition Kit DSST can be integrated into a suite of clinical measures within a single app, allowing multiple clinically relevant assessments to be performed. For example, depressive symptom, functioning, and quality of life measures may be offered alongside medication adherence and adverse event reporting, as well as reminders.

The THINC-it Tool has been previously validated as a screening tool and as a repeat measure for cognitive function in adults with MDD [12,23]. A variant of the DSST was included in the THINC-it Tool and accounted for significant variance in the tool’s overall performance, suggesting that the DSST alone may provide sufficient conceptual coverage [12,23,24]. This study demonstrated the validity of the app-based Cognition Kit DSST to assess cognitive impairment in patients with MDD and represents a personalized assessment approach that may help guide MBC to inform psychotherapeutic and pharmacologic treatment options.

Moreover, applying smartphone technology may help clinicians to more fully understand the mediational role of cognition in MDD, particularly the extent to which it interferes with daily life in patients with persistent psychosocial and workplace impairment [6]. Integrating cognitive functions as part of the assessment of MDD may inform suicide risk, as suicidality in some cases may be linked to cognitive function [25]. Therefore, there is a need to assess cognition in these patients and in patients who do not functionally recover and will continue to exhibit cognitive impairment despite treatment. Deploying an easily accessible smartphone-based testing regimen that can be completed outside the clinical environment at no cost and with limited inconvenience to the patient may help remove barriers to routine assessment.

There is a risk, however, that assessments performed using a paper-and-pencil approach may differ compared with a smartphone-based assessment. The DSST is a polyfactorial test that assesses motor speed, attention, and visuoperceptual functions, all of which may be subject to subtle inter- and intraindividual differences when evaluated using a paper-and-pencil approach versus a smartphone-based approach [16]. However, high test-retest reliability has previously been demonstrated with the paper-and-pencil version [26]. DSST performance is known to be reduced with increasing age and may be negatively influenced by physical impairments relating to vision or motor skills, but level of education does not appear to significantly influence performance [27]. Women may also perform better than men [28]. Therefore, additional neurocognitive testing may be required alongside the DSST to confirm any deficit.

Preliminary evidence suggests that there are differences among antidepressants in their ability to affect cognitive function in patients with MDD [5], indicating that cognitive function assessment is highly relevant when initiating pharmacologic therapy. In fact, DSST has been effectively used to assess improved performance in patients with MDD when comparing 2 different antidepressants [5,29]. That the Cognition Kit DSST was able to detect cognitive dysfunction in patients with MDD demonstrates that it may be implemented into an MBC framework because it is capable of guiding treatment decision-making for clinicians.

Implementing the Cognition Kit DSST in routine clinical practice has several benefits for both clinicians and patients. Enabling patients to complete neurocognitive assessment tools on their smartphones conserves clinical resources and streamlines the assessment process. For example, having a preassessed electronic record of cognitive symptom status can improve testing accuracy and consistency, while enabling clinicians to focus appointment time on treatment as opposed to administering and interpreting paper-and-pencil versions of cognitive assessments [30]. Furthermore, patients can easily and privately access app-based assessments at their convenience. Currently, assessment is recommended every 2-4 weeks if clinically appropriate, but patients could complete the Cognition Kit DSST more frequently, if required, to build a rich longitudinal picture of their cognition during a clinical study or as part of a health care pathway. However, when deploying these electronic assessments, consideration needs to be given to applying appropriate data protection measures to ensure patient privacy [30], especially given that individuals with depression may be concerned about employers, for example, becoming aware of a diagnosis of MDD or a degree of cognitive impairment [30].

This study had several strengths, including the patient population being representative of patients with MDD based on MADRS score, diagnosis confirmed by the MINI, and anxiety and anhedonia assessed by HAM-A and SHAPS, respectively. Furthermore, patients with heterogeneous illness presentation and course were eligible, including those receiving psychotropic medications in combination with medications for concurrent comorbidities.

### Limitations and Future Directions

Although the app-based Cognition Kit DSST was validated in patients with MDD, it still needs to be evaluated in healthy controls and individuals with MDD in other settings (eg, primary care). Additionally, Cognition Kit DSST sensitivity to change remains to be assessed. Our study excluded patients whose principal diagnosis was not MDD, which was another limitation. Further, only one standardized measure of cognitive function was validated in a relatively small sample, and patients were recruited from treatment-resistant depression centers. These factors may limit the generalizability of study results to a broader patient population, including those with treatment-responsive MDD or those who are treatment naive. Likewise, these results may not be generalizable to individuals with neurological disorders other than MDD, such as dementia, or those with learning differences, such as dyslexia.

The DSST has been extensively studied and is well regarded as a multidomain assessment of cognitive function [16]; however, it may disproportionately evaluate processing speed, and therefore, may not provide adequate conceptual coverage of other subdomains of neurocognition. Furthermore, the Cognition Kit DSST does not contain any self-reported measures of cognitive function, and we acknowledge that self-reported cognitive function does not correlate with objective cognitive function.

### Conclusions

This study demonstrated that cognitive function assessments performed using the Cognition Kit DSST app correlated with the paper-and-pencil version of the test, detecting cognitive deficits in adults with MDD. Future research efforts should focus on validating the Cognition Kit DSST in a healthy control population and in a larger MDD patient population. Research is needed into the Cognition Kit DSST app’s sensitivity to change with treatment, the cost-effectiveness and impact on therapeutic outcomes of implementing the app, as well as the app’s implications for health outcomes.

## Appendix

Multimedia Appendix 1 Evaluation of an app-based Digit Symbol Substitution Test for assessment of cognitive deficits in adults with major depressive disorder.

## Abbreviations

DSST: Digit Symbol Substitution Test
HAM-A: Hamilton Anxiety Rating Scale
MADRS: Montgomery-Åsberg Depression Rating Scale
MBC: measurement-based care
MDD: major depressive disorder
MINI: Mini-International Neuropsychiatric Interview
PROs: patient-reported outcomes
SHAPS: Snaith-Hamilton Pleasure Scale
