# Supplementary material for: An App-Based Digit Symbol Substitution Test for Assessment of Cognitive Deficits in Adults With Major Depressive Disorder: Evaluation Study
Source: JMIR Ment Health. 2022 Oct 27;9(10):e33871. doi: 10.2196/33871 (PMC9650567; doi:10.2196/33871)
Supplement: Multimedia Appendix 1 [file mental_v9i10e33871_app1.pdf]

Supplementary Material for:

Evaluation of an App-Based DSST for Assessment of Cognitive Deficits in Adults With MDD

McIntyre RS, et al.

Supplementary Figure. Patient disposition.

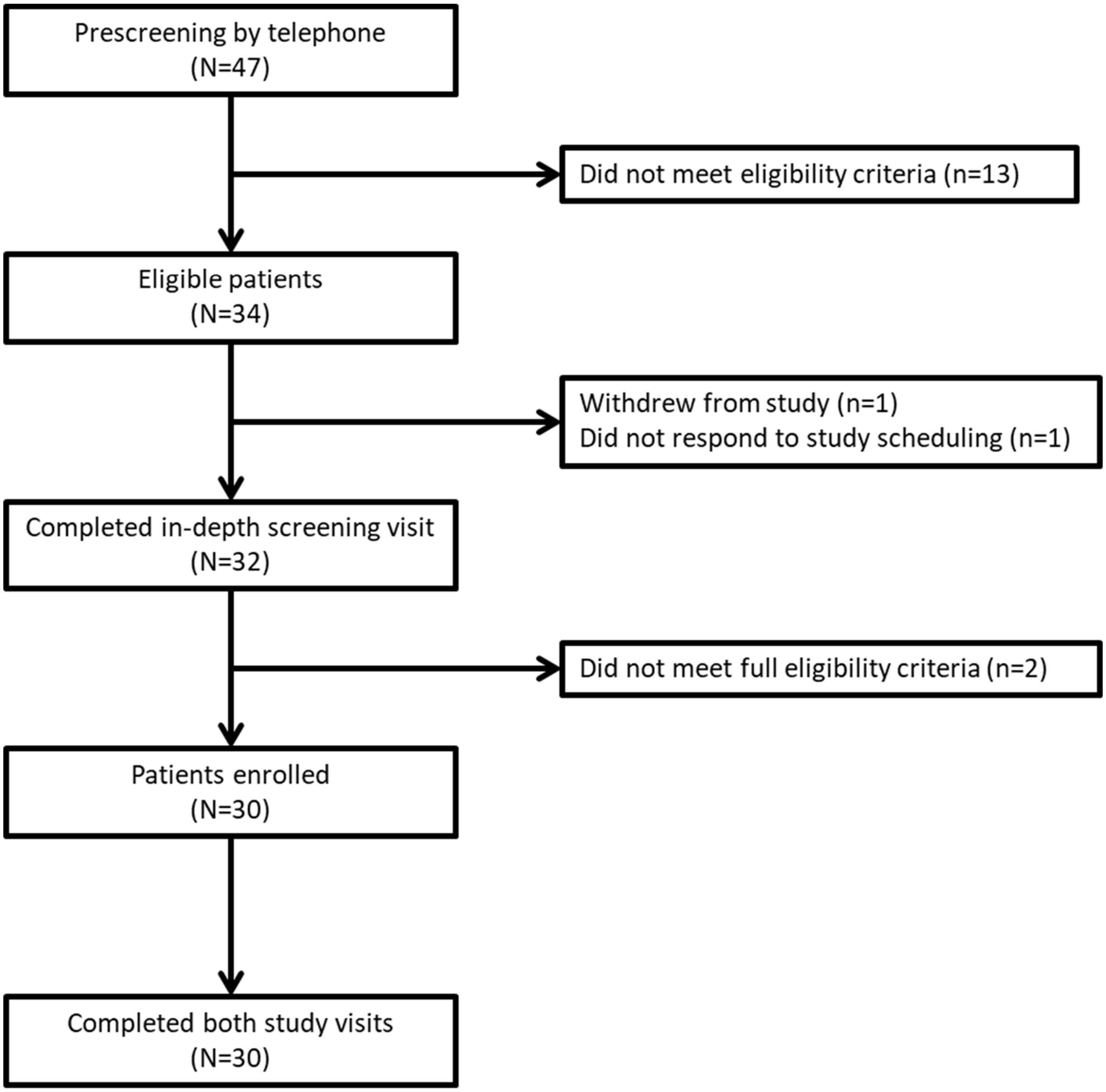

Prescreening was performed for 47 potential patients, of which a total of 30 patients were eligible for inclusion.
